# Uncovering Household Tuberculosis Infection Testing and Care Patterns Using a Novel Bioinformatics Linkage Strategy

**DOI:** 10.1093/cid/ciag079

**Published:** 2026-02-11

**Authors:** Jeffrey I Campbell, Meredith B Brooks, Jenine Dankovchik, Sophia Giebultowicz, Angelica Falkenstein, Heather Angier, C Robert Horsburgh, Cynthia A Tschampl, Jessica E Haberer, Jonathan Bressler, Rachel L Epstein, Helen E Jenkins, Karen R Jacobson

**Affiliations:** Section of Pediatric Infectious Diseases, Department of Pediatrics, Boston Medical Center, Boston, Massachusetts, USA; Department of Global Health, Boston University School of Public Health, Boston, Massachusetts, USA; OCHIN, Portland, Oregon, USA; OCHIN, Portland, Oregon, USA; OCHIN, Portland, Oregon, USA; Department of Family Medicine, Oregon Health & Science University, Portland, Oregon, USA; Department of Global Health, Boston University School of Public Health, Boston, Massachusetts, USA; Department of Epidemiology, Boston University School of Public Health, Boston, Massachusetts, USA; The Heller School for Social Policy and Management, Brandeis University, Waltham, Massachusetts, USA; Center for Global Health, Massachusetts General Hospital, Boston, Massachusetts, USA; Department of Pediatrics, Boston Medical Center, Boston, Massachusetts, USA; Section of Pediatric Infectious Diseases, Department of Pediatrics, Boston Medical Center, Boston, Massachusetts, USA; Section of Infectious Diseases, Department of Medicine, Boston Medical Center, Boston, Massachusetts, USA; Department of Biostatistics, Boston University School of Public Health, Boston, Massachusetts, USA; Section of Infectious Diseases, Department of Medicine, Boston Medical Center, Boston, Massachusetts, USA

**Keywords:** latent tuberculosis infection, household, care cascade, tuberculosis preventive therapy

## Abstract

**Background:**

Households are a focus of tuberculosis (TB) active case finding strategies. However, little is known about patterns of (noninfectious) TB infection clustering in households without a known infectious index case, or how household membership affects progression through the TB infection care cascade.

**Methods:**

Using data from a multistate community health center network in the United States, we identified individuals with a positive tuberculin skin test (TST) or interferon gamma release assay (IGRA) between 2014 and 2022. We implemented an algorithm to link these “sentinel patients” with household members in the database. We determined rates and predictors of TST/IGRA testing, test positivity, and treatment prescription among household members.

**Results:**

We identified 35 772 sentinel patients with a positive TST or IGRA, who were linked to 129 432 household members. Of household members, 33 821 (26.1%) had a TST/IGRA within 2 years of the sentinel patient's positive test, of whom 3127 (9.3%) had a positive test and 641 (20.6%) were prescribed TB infection treatment. Whether the sentinel patient was prescribed treatment was associated with household member being tested (adjusted odds ratio [aOR] 1.16 [95% confidence interval {CI}: 1.10–1.21]) and being prescribed treatment (aOR 9.68 [95% CI: 7.71–12.16]).

**Conclusions:**

Most household members had no documented TB infection test before or within 2 years after a sentinel patient in the household had a positive test. Household member and sentinel patient characteristics, conditions, and treatment were associated with household member testing, test positivity, and treatment prescription. Households may be an effective but underutilized context to identify and treat individuals with TB infection, even when no TB disease cases are present.

## Teaser

Using a bioinformatics linkage strategy in a large clinical database, we found high rates of tuberculosis (TB) infection test positivity among parents of children with TB infection and partners of individuals with TB infection. Tuberculosis infection treatment clustered within households.

## BACKGROUND

Anestimated 8–13 million individuals in the United States have latent tuberculosis (TB) infection (henceforth: “TB infection”), and more than 10 000 people were diagnosed with TB disease in 2024 [1–4]. Targeted testing approaches to identify individuals with TB infection rely upon screening for individual risk factors, including birth outside the United States, exposure to an individual with infectious TB disease, and conditions associated with increased progression to TB disease [5]. Recent studies have found low rates of testing among individuals with risk factors [6–8]. Gaps in TB infection screening and treatment contribute to the ongoing burden of TB in the United States, where 80% of TB disease cases arise from reactivated TB infection [9] and only 10% of those with TB infection complete preventive treatment [2]. Consequently, most patients who are diagnosed with TB disease in the United States may have missed an opportunity for prevention. Better strategies to identify, test, and treat individuals at highest risk for TB infection are needed to achieve TB elimination in the United States [10].

Onepotential context for improving TB infection screening and treatment is the household. Household contact tracing is a pillar of TB disease detection, but households are not routinely ascertained—and household members are not routinely tested—when individuals are diagnosed with TB infection rather than disease. Although TB testing is recommended for close contacts of children with a positive tuberculin skin test (TST) or interferon gamma release assay (IGRA) to identify potential source cases with infectious TB disease [11], close contact testing has not been used to enhance TB infection diagnosis as a goal unto itself. Furthermore, while qualitative studies suggest that social support, TB stigma, and TB knowledge and beliefs among family members may be important drivers of TB infection care engagement [12, 13], these effects have not been directly measured.

Understanding household patterns of TB infection diagnosis and care engagement could inform new household and family-centered approaches to TB infection testing and treatment. We adapted and implemented a bioinformatics strategy to link individuals in inferred household units within a US community health center network. Our objective was to identify factors associated with TB infection testing, diagnosis, and treatment among household members after an individual in the household had a positive TB infection test (the “sentinel patient”).

## METHODS

We conducted a retrospective cohort study of patients with a positive TB infection test and their household members receiving care in the OCHINnetwork [14]. OCHIN is a nonprofit organization that provides a centralized electronic health record (EHR) to US community health organizations, which collectively include >1200 clinical sites in 40 states. Sites include federally qualified health centers, community health centers, and public health departments, among others. Individual patient data are harmonized and housed in OCHIN's Research Data Warehouse [15].

### Identifying Sentinel Patients With Tuberculosis Infection and Linking Them to Household Members

We identified all patients with positive TB infection tests (TST or IGRA) between 2014 and 2022 who had at least 2 clinical encounters in the study timeframe. We linked these individuals to household members with clinical information available in the data warehouse (Supplementary Figure 1). Briefly, to identify household, social, and family contacts of patients with TB infection, we used 4 data elements within the data warehouse: a mother–child linkage table, a patient relationships table, insurance coverage account data, and address history records. We inferred contacts directly from the mother–child and patient relationships tables. For insurance data, we identified any patient who shared a health insurance account or address with an individual with a positive test. We excluded group insurance account types (eg, corporate and employer) and accounts shared by a high number of patients, because these were unlikely to reflect meaningful connections. For address histories, we cleaned and standardized addresses and identified patients who shared the exact address or geocoded coordinates with an individual with a positive test during overlapping time periods. We identified apartment buildings as addresses shared by more than 12 patients with at least 3 units and group quarters (eg, shelters) as addresses shared by more than 12 patients within 1 unit. Unit-level matching was required for apartments. Insurance data linkages in the OCHIN database have been previously validated [16]. We assumed for this analysis that contemporaneous shared unit-level addresses represented socially and epidemiologically relevant shared household membership. Patient-reported relationships were extracted from patient relationships tables and insurance coverage account data (Supplementary Table 1).

We defined the sentinel patient to be the first (chronological) patient within the household to have a positive TB infection test. When multiple individuals had positive TB infection tests on the same day, we randomly assigned 1 to be the sentinel patient and others to be household members; we performed a sensitivity analysis around this assignment. Once sentinel patients were identified, we merged data on all household member TB infection testing between 2012 and 2024 regardless of result, thus bracketing all sentinel patients' positive tests by at least 2 years. To focus this analysis on TB infection rather than TB disease, we excluded sentinel patients and their household members when the sentinel patient had TB disease, or when a household member was diagnosed with TB disease (in the absence of a previous positive TST/IGRA) before or on the same day as the sentinel patient's first positive test. We excluded sentinel patients whose first positive test was obtained before any household member was born, and household members who were born after the sentinel patient had their first positive test. We also excluded sentinel patient–household member pairs linked only through shared group housing (eg, homeless shelters).

### Outcomes

The primary outcome was whether a linked household member received a TST or IGRA from any time before to within 2 years after the sentinel patient's first positive test. Secondary outcomes were (1) presence of a positive TST or IGRA among linked household members who were tested within 2 years of the sentinel patient's first positive test and (2) household member TB infection treatment prescription within 1 year after the household member's positive test.

### Exposures

We examined testing, test positivity, and treatment prescription patterns within specific sentinel patient–household member relationships. We examined additional exposures drawn from literature on barriers to TB infection care in the United States [6, 17, 18], including individual-level demographic characteristics (age, sex, and language preference), household-level social vulnerability index (SVI) quartile [19], and medical characteristics (human immunodeficiency virus [HIV], other immunocompromising conditions, and other comorbidities [diabetes and kidney and liver diseases]) (Supplementary Table 2). Because country of origin was missing for most patients, we excluded this exposure from the main analysis but included this variable in sensitivity analyses.

### Analysis

We used descriptive statistics to summarize sentinel patient and household member cohorts. We used univariable and multivariable logistic regression to assess the relationship between exposures and outcomes, using a cluster term for households to account for nonindependence between members of each household. We represent results as odds ratios and depict the primary analysis using marginal predicted probabilities for each variable and outcome.

We performed sensitivity analyses. First, because the main analysis randomly assigned 1 patient to be a sentinel and others to be household members when 2 or more patients tested positive on the same day, we conducted an analysis in which we removed all sentinel patient–household member pairs with positive tests on the same day. Second, to liberalize the timeframe in which household members could be tested, we analyzed household member testing at any time before or after the sentinel patient's positive test (ie, not restricted to testing within 2 years after the sentinel patient tested positive). Third, because shared address defined most linkages, we performed analyses restricted to relationships established by (1) address links and (2) nonaddress links. Fourth, we conducted a sensitivity analyses to assess the effect of self-reported birth in countries with elevated TB incidence (defined as >10 TB cases/100 000 population/year [20]), using (1) a complete case analysis and (2) multiple imputation of sentinel and household member birth in a high-incidence country, using chained models that incorporated exposure and outcome variables plus census tract proportion of individuals who were born outside of the United States. Fifth, we analyzed the effects of multilevel clustering by developing hierarchical mixed effects models accounting for clustering at the sentinel patient level, clustered within the clinics where each sentinel patient was tested. Finally, to mitigate the possibility that our findings reflected public health contact tracing, we additionally excluded all sentinel patients and household members with a diagnostic code for TB exposure, and/or who were tested in public health, TB, or infectious disease clinics.

The Boston University Medical Campus IRB deemed this study exempt (H-45013). Analysis was performed in Stata v19 (StataCorp, College Station, Texas) and *R* version 4.4.3.

## RESULTS

**Figure 1.**
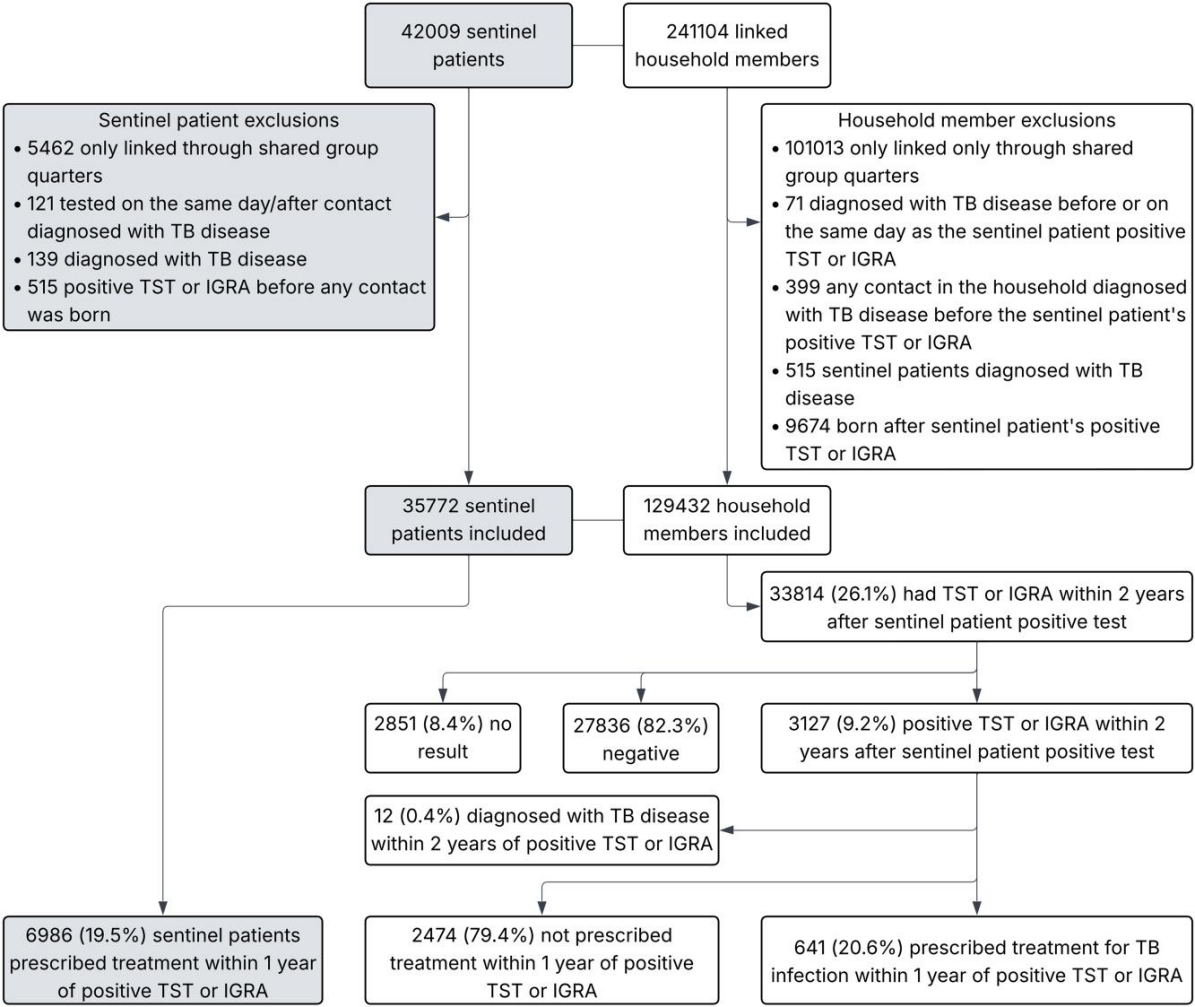
Consort diagram and care cascade outcomes for household members. Abbreviations: IGRA, interferon gamma release assay; TB, tuberculosis; TST, tuberculin skin test.

**Figure 2.**
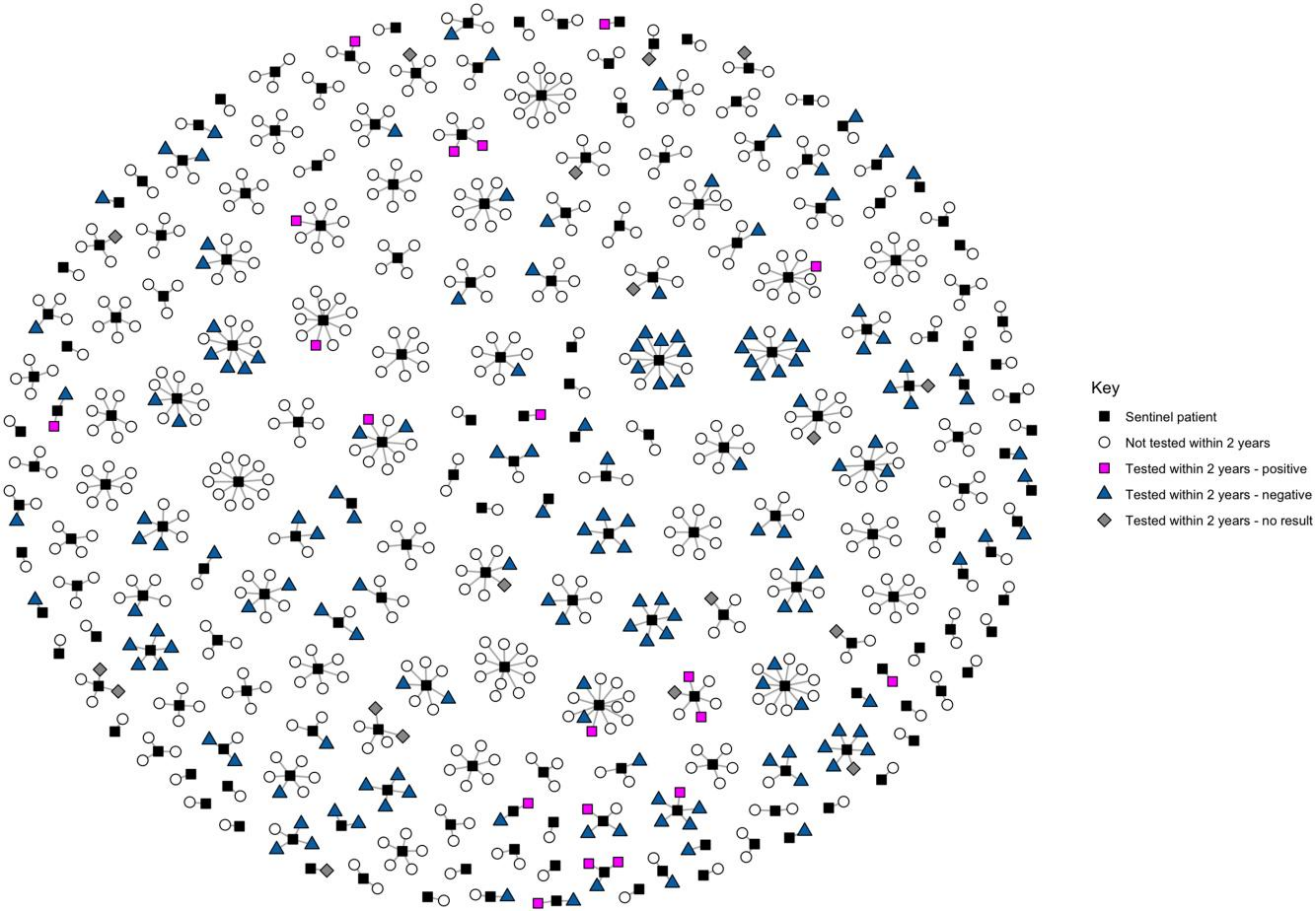
Household membership networks for a random sampling of 200 sentinel patients and their household members.

A total of 42 009 sentinel patients were linked to 241 104 household members; after applying exclusions, there were 35 772 sentinel patients and 129 432 household members included in the primary analysis (Figure 1; Table 1). Sentinel patients had a median of 3 (interquartile range [IQR] 1–4) household members. Figure 2 illustrates a sample pattern of testing and test positivity within household networks.

**Table 1.** Initial Patient and Household Member Characteristics

Characteristic	Sentinel Patients (N = 35 772)	Household Members (N = 129 432)
Sex		
Male	22 501 (62.9%)	71 663 (55.4%)
Female	13 268 (37.1%)	57 742 (44.6%)
Missing	3 (<1%)	27 (<1%)
Age at time of sentinel patient diagnosis (y)		
<5	616 (1.7%)	11 698 (9.0%)
5–15	1724 (4.8%)	26 608 (20.6%)
16–24	4231 (11.8%)	23 254 (18.0%)
25–44	13 999 (39.1%)	36 084 (27.9%)
45–64	10 692 (29.9%)	23 264 (18.0%)
65+	3633 (10.2%)	8524 (6.6%)
Missing	877 (2.5%)	0 (0.0%)
Language preference		
English	10 819 (30.2%)	50 163 (38.8%)
Spanish	13 625 (38.1%)	44 490 (34.4%)
Haitian Creole	446 (1.2%)	1251 (1.0%)
Other	10 849 (30.3%)	32 632 (25.2%)
Missing	33 (0.1%)	896 (0.7%)
Country of origin		
Elevated TB incidence	1017 (2.8%)	6599 (5.1%)
Not elevated TB incidence	8674 (24.2%)	21 220 (16.4%)
Missing	26 081 (72.9%)	101 613 (78.5%)
Social vulnerability index		
Quartile 1 (least vulnerable)	2155 (6.0%)	6833 (5.3%)
Quartile 2	4449 (12.4%)	14 876 (11.5%)
Quartile 3	8076 (22.6%)	29 320 (22.7%)
Quartile 4 (most vulnerable)	19 159 (53.6%)	71 457 (55.2%)
Missing	1933 (5.4%)	6946 (5.4%)
Comorbidities		
HIV	588 (1.6%)	641 (0.5%)
Other immunocompromising conditions	1332 (3.7%)	3009 (2.3%)
Other comorbidities	6780 (19.0%)	12 671 (9.8%)
Relationship of household member to sentinel patient		
Child	NA	29 328 (22.7%)
Sibling	NA	2199 (1.7%)
Parent (sentinel patient 0 to <5 y old)	NA	465 (0.4%)
Parent (sentinel patient 5–14 y old)	NA	1173 (0.9%)
Parent (sentinel patient 15+ years old)	NA	8259 (6.4%)
Partner	NA	669 (0.5%)
Other relative	NA	564 (0.4%)
Shared household (relationship not otherwise specified)	NA	86 767 (67.0%)
Missing	NA	8 (0.0%)
Outcomes		
Tested for TB infection within 2 y of sentinel patient	NA	33 814 (26.1%)
Positive TST or IGRA	35 722 (100%)	3127 (9.2% of those with tests)
Started treatment within 1 y of positive test	6986 (19.5%)	641 (20.6% of those with positive tests and no TB disease)

Abbreviations: IGRA, interferon gamma release assay; HIV, human immunodeficiency virus; NA, not applicable; TB, tuberculosis; TST, tuberculin skin test.

### Care Cascade

Of the 129 432 household members included, 33 814 (26.1%) were tested at a time before to within 2 years after the sentinel patient's first positive test. An additional 12 291 (9.5%) had their first test 2 or more years after the sentinel patient's first positive test. Of the 33 814 household members who had a TB infection test, 3127 (9.2%) tested positive. Of patients with positive tests, 12 (0.4%) were diagnosed with TB disease within 2 years, none of whom had been prescribed TB infection treatment. Of the 3115 household members with a positive test and no incident TB disease, 641 (20.6%) were prescribed TB infection treatment within 1 year of testing positive. Supplementary Figures 2–4 illustrate timing of cascade steps.

### Patterns of Household Member Testing

**Figure 3.**
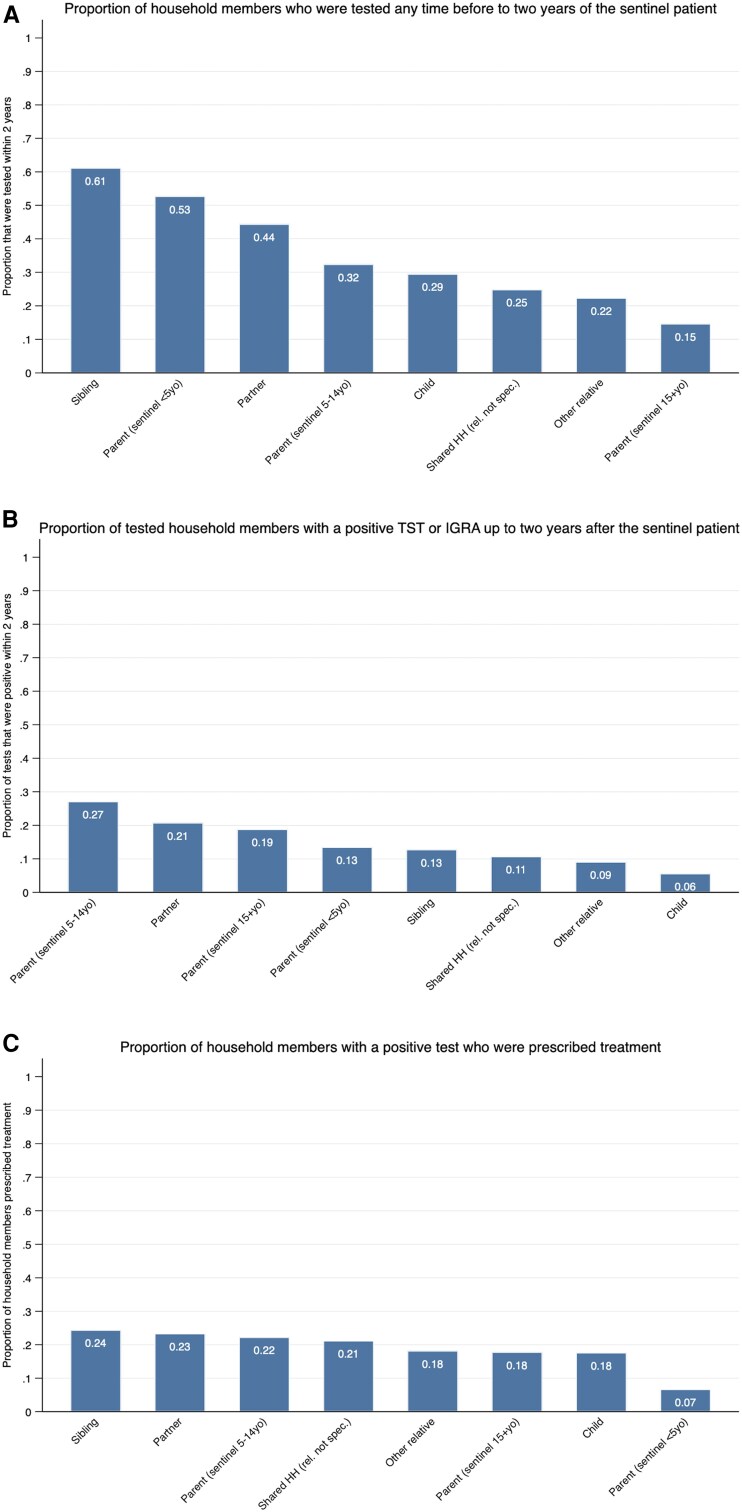
*A*–*C*, Proportion of household members who were tested from any time before to 2 y after the sentinel patient's first positive test (*A*), household member test positivity (positive test if tested) within 2 y of the sentinel (*B*), and household members who started treatment within 1 y of a positive test (*C*), by relationship to the sentinel patient. Abbreviations: rel. not spec., “relationship not specified”; yo, years old.

**Figure 4.**
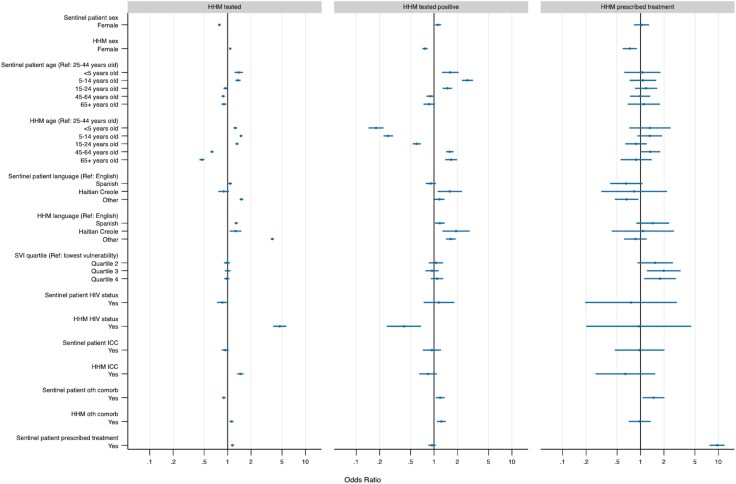
Characteristics of initial patients, household members, and households associated with household member TST or IGRA testing, test positivity, and treatment prescription. Abbreviations: ICC, immunocompromising condition; IGRA, interferon gamma release assay; incid. Crty, “incident country”; HHM, household member; Ref, reference; TB, tuberculosis; TST, tuberculin skin test.

Siblings had the highest rates of testing (61%), and parents of children aged 15 or older had the lowest (15%) (Figure 3*A*). In multivariable analysis, both sentinel patient and household member demographic characteristics and medical factors were associated with odds of household member testing (Figure 4; Supplementary Table 3 and Figure 5). Sentinel patient treatment prescription was associated with increased adjusted odds of household member testing (adjusted odds ratio [aOR]1.16 [95% confidence interval (CI): 1.10–1.21]).

### Patterns of Household Member Test Positivity

Parents of sentinel patients 5–14 years old (27%) and ≥15 years old (19%), and partners of sentinel patients (21%), had the highest test positivity (Figure 3*B*). Figure 4 (Supplementary Table 4) shows sentinel patient and household member characteristics associated with household member test positivity, among those household members who were tested. We found that younger age of sentinel patients was associated with increased odds of a household member testing positive, whereas younger age of household members was associated with decreased odds of testing positive. Household member HIV was associated with decreased adjusted odds of having a positive test (aOR 0.41 [95% CI: .25–.68]).

### Patterns of Household Member Tuberculosis Infection Treatment Prescription

The proportion of household members with positive tests who were prescribed treatment was similar across most relationship categories, though parents of sentinel patients <5 years old had low rates of prescription (7%). In multivariable analysis, sentinel patient TB infection treatment was highly associated with a household member also being prescribedTB infection treatment (aOR 9.68 [95% CI: 7.71–12.16]) (Figure 4; Supplementary Table 5 and Figure 5). Higher household SVI quartile (ie living in a neighborhood with more vulnerability) was also associated with higher odds of TB infection treatment prescription.

### Sensitivity Analyses

Sensitivity analyses yielded similar results to the main analysis (Supplementary Figures 6–13). In the complete case analysis including sentinel patient and household member birth country, we found that household members with known birth in a country with elevated TB incidence had increased odds of being tested (aOR 2.44 [95% CI: 2.18–2.74]) and testing positive (aOR 1.36 [95% CI: 1.01–1.76]) (Supplementary Figure 10). In the analysis using rigorous exclusion of known TB disease contact, the proportion of household members tested, yield of testing, and proportion prescribed treatment was slightly lower than in the main analysis (Supplementary Figure 12).

## DISCUSSION

Using a novel household linkage approach, we characterized key TB infection care cascade steps among household members after a sentinel patient in the household had a positive TST or IGRA. We identified household member groups with high TB infection test positivity rates, including parents of sentinel patients ≥5 years old and partners of the sentinel patient. We also found evidence that TB infection treatment uptake is influenced by others in the household starting treatment. US TB guidelines for adults and children recommend testing individuals with risk factors for TB infection and treating those diagnosed [5]; diagnosing and treating individuals with TB infection is a core component of the TB elimination strategy in the United States [21]. Our results suggest that households may be important units to target for TB infection testing and treatment, even when no individual with infectious TB is identified in the household.

We found differences in testing and test positivity by characteristics of household members that likely reflect use of current targeted testing strategies and known higher burden of TB infection among specific groups. As previously observed [22], we found increased testing but low positivity among people with HIV, which likely arises from protocolized testing of this population [23] who may otherwise have low risk for TB infection. We also found increased testing but lower test positivity among female household members. Population-based studies have estimated that males in the United States have higher burden of TB infection and disease [24, 25]. Our findings add to evidence that males may be relatively under-screened despite their increased risk [22]. We found that birth in a country with elevated TB incidence was associated with increased testing and test positivity.

More surprising were the associations between sentinel patient characteristics and household member testing, test positivity, and treatment prescription. Household members of child sentinel patients had higher odds of being tested and of having a positive TB infection test than household members of adult sentinel patients. The American Academy of Pediatrics and National TuberculosisCoalition of America recommend testing household members of young children with TB infection to identify source cases [11, 26], even though sources are rarely detected [27]. In our study, when the sentinel patient was <5 years old, we found that less than half of household members had been tested within 2 years, but of those tested, 13% had a positive test. We found that parents of sentinel patients ≥5 years old children had even higher test positivity rates—19%–27%—although older children with TB infection do not routinely prompt source case detection efforts. These patterns of positive testing within households likely signify shared risk factors and shared but undetected exposures to TB.

We found that initial patient treatment prescription was associated with household member testing and TB infection treatment prescription. The household member TB infection treatment prescription rate (20.6%) in our study is lower than prescription rates in other recent studies, including Kaiser Permanente patients in California (33%) [28] and patients receiving care at US public health TB clinics (42%) [18]. This discrepancy may be due to different prescribing patterns among community health center providers, differences in TB infection classification, and differences in patient populations. Regardless, the low rates seen in each of these studies suggests substantial under-treatment of at-risk patients. A 2016 TB infection care cascade meta-analysis [29] and subsequent care cascade analyses in US populations [6, 17, 18] showed steep drop-offs between TB infection diagnosis and treatment prescription. Survey and qualitative studies of patients diagnosed with TB infection have identified multiple patient-level factors that lead to treatment refusal, including TB stigma, skepticism toward the diagnosis, and lack of knowledge [12, 30, 31]. Structural barriers to accessing care—such as distance to clinics—as well as clinician knowledge gaps, concerns about medication toxicity, and determination that tests were falsely positive also contribute to nonprescription [32]. Notably, we found that odds of treatment prescription were higher for household members living in areas with higher social vulnerability, as measured by the SVI. Several mechanisms could underlie this result, such as increased access to clinicians with experience treating TB infection in these areas, or interaction between social vulnerability and clinic proximity [33]. The combined effects of TB-related attitudes and ability to access care likely cluster within households, leading to the observed correlated levels of care engagement in sentinel patients and household members. Interventions aimed at improving TB infection care have largely sought to address individual- or health system-level barriers to care [34–36]. Interventions aimed at household-level facilitators of care engagement, such as household education interventions, may augment these efforts.

To perform this study, we used a bioinformatics linkage strategy that may be useful for future TB and other infectious disease research. Household linkage algorithms are being increasingly incorporated into EHR and claims databases [37]. The capacity to discern relational networks in these databases holds promise to examine a range of behavioral and epidemiologic patterns related to infection clustering and transmission [38]. The integration of laboratory, prescription, demographic, and administrative data alongside household linkages, as we did in this study, could enhance infectious disease research within clinical and administrative databases that increasingly include relational ties. Linkages within clinical databases could also facilitate household-level clinical and public health interventions. Implementation of such linkage techniques in clinical and public health programs would need to carefully consider how and withwhom data would be shared, and how household members would be identified and approached, to facilitate public health benefit while also protecting patient privacy and autonomy (Supplementary Figure 14).

Our study has limitations. First, limitations of our household linkage mechanism included inferred rather than directly measured household membership for most linkage strategies (eg, shared address), assumptions that household membership corresponded to clinically or epidemiologically relevant interactions, and lack of ability to identify household members receiving care outside the OCHIN network. Ongoing work is seeking to understand the overlap of self-reported and EHR-derived linkage networks, to provide additional external validation of linkage procedures. Second, our TST and IGRA positivity findings rely upon routine clinical testing of household members. Because most household members were not tested, the true TB infection prevalence among household members could not be determined. Third, we used a community health network EHR database that may not have captured data for patients receiving components of care outside the network, such as in public health TB clinics. Fourth, while US community health centers serve a large population at risk for TB infection, our findings may not generalize to individuals receiving primary care in other settings, or who are unable to access primary care. Fifth, we were not able to glean nuanced medical decision-making about treatment recommendations, such as adjudication of false positive tests or decisions to recommend against treatment due to comorbidities. Sixth, we used data derived primarily from structured health record fields and had limited ability to account for missing or unstructured data. Notably, although birth outside the United States is an important risk factor for TB infection, data on birth countries were missing for three quarters of patients. Seventh, we could not definitively classify risk factors for TB infection or indications for testing, such as TB exposure or extended travel to high incidence countries. Finally, it is possible that sentinel patients and household members were tested in the context of contact tracing after exposure to individuals with TB disease who were outside the OCHIN network; our exclusion of households with known TB disease, and additional sensitivity analyses, reduces but does not fully eliminate this possibility.

In conclusion, we found variable rates of household member testing after a sentinel patient had a positive TST or IGRA. We found high rates of test positivity among household members of children and adolescent sentinel patients. Tuberculosis infection treatment for household members was highly associated with whether the sentinel patient was prescribed treatment. Household-level TB infection engagement strategies may improve testing and treatment uptake to advance TB elimination goals.

## Supplementary Material

ciag079_Supplementary_Data

## Supplementary Data

Supplementary materials are available at *Clinical Infectious Diseases* online. Consisting of data provided by the authors to benefit the reader, the posted materials are not copyedited and are the sole responsibility of the authors, so questions or comments should be addressed to the corresponding author.
