# Supplementary material for: Uncovering Household Tuberculosis Infection Testing and Care Patterns Using a Novel Bioinformatics Linkage Strategy
Source: Clin Infect Dis. 2026 Feb 11;83(1):e167–76. doi: 10.1093/cid/ciag079 (PMC13180345; doi:10.1093/cid/ciag079)
Supplement: ciag079_Supplementary_Data [file ciag079_supplementary_data.docx]

**APPENDIX**

**SUPPLEMENTAL METHODS**

**Detailed Household Linkage Methods**

1. **Family Linkage Table (Mother-Child Pairs)**
   1. **Data Source Description**

The Family Linkage table contains identified mother-child pairs within the Research Data Warehouse, refreshed weekly. OCHIN Research developed this process which relies on several sources and methods including explicit linkages (coverage accounts, emergency contacts, OB claim forms) and imputed linkages (shared Medicaid case numbers, addresses, home phone numbers, and fuzzy matching of emergency contact demographics). Although these methods are similar to those applied in our broader household linkage process, the mother-child linkages were established independently prior to this project.

- 1. **Extraction**

We extracted all mother-child pairs where either the mother or child was in our index patient cohort. Using the mother’s patient ID yielded **60,807 relationships for 25,190 unique index patients and 60,807 unique contact patients**. Using the child’s ID yielded **20,613 relationships for 20,613 unique index patients and 18,778 unique contact patients.**

- 1. **Characteristics**

All relationships from this table were coded as mother-child and treated as static (non-temporal). After deduplication, this source contained **81,420 relationships for 39,887 unique index patients and 79,418 unique contact patients.**

1. **Patient Relationships Table**
   1. **Data Source Description**

This table contains significant relationships recorded in the patient’s chart, including but not limited to emergency contacts, with an explicit relationship type. Potential relationship types included:

- Spouse/partner (aggregate of spouse, significant other, and partner)
- Parent (aggregate of mother, father, parent)
- Step parent (aggregate of step mother, step father, step parent)
- Step child (aggregate of step daughter, step son, step child)
- Child (aggregate of daughter, son, child)
- Relative
- Friend/confidential contact
- Sibling (aggregate of sister, brother, sibling, step sister, step brother)
- Aunt
- Uncle
- Niece
- Nephew
- Grandparent (aggregate of Grandmother, Grandfather, Grandparent)
- Grandchild (aggregate of Granddaughter, Grandson, Grandchild)
- Son-in-law
- Daughter-in-law
- Parent-in-law
- Cousin
- Foster parent
- Foster child/ward
- Other guardian
- Ex-spouse
- Sponsor/sponsee
- Caregiver
- Caseworker
- Intensive home service provider
- Client/patient/ward
- Religious
- Roommate
- Neighbor
  1. **Extraction**

We extracted all rows for any index cohort patient, retaining the relationship type (i.e., the index patient’s relationship to the contact). We then generated the reciprocal relationship from the contact’s perspective (e.g., if the index patient was listed as the contact’s parent, we classified the contact patient as a child of the index patient). The initial extraction yielded **2,324 relationships for 2,110 unique index patients and 2,162 unique contact patients.**

- 1. **Cleaning**

We deduplicated these relationships against the set from the Family Linkage table, retaining one row per relationship while preserving information from both sources. Where there were discrepancies, we retained the more specific information preferentially (e.g., if one source showed “parent-child” as the relationship type and other “mother-daughter” as the relationship type, we retained the latter). In rare cases where there was no obvious “correct” relationship and a discrepancy existed; we reviewed demographics manually to determine the most accurate classification.

- 1. **Characteristics**

Relationships from this table were treated as static and did not require address matching. After deduplication, the combined relationship table (Family Linkage and Patient Relationship sources) contained **82,869 relationships for 40,516 unique index patients and 80,577 unique contact patients.**

1. **Insurance Coverage Account Table**
   1. **Data Source Description**

This table contains one row per coverage account a patient has registered in their chart, as well as the patient’s relationship to the guarantor on the account.

- 1. **Extraction**

We first extracted all coverage accounts for any index cohort patient, then extracted all additional patients sharing those account IDs, generating a set of potential linkage pairs based on shared insurance accounts. The initial extraction yielded **7,235,149 relationships for 22,129 unique index patients and 133,044 unique contact patients.**

- 1. **Cleaning**

To limit these relationships to epidemiologically meaningful links:

- - 1. We excluded certain account types we found did not reliably reflect meaningful household or social connections (e.g., Corporate, Employee, Third-Party Liability).
    2. We excluded accounts shared by an unusually high number of patients as these likely reflect administrative groupings rather than true household or social connections. We determined the patient count thresholds by iteratively examining the names and addresses of shared members until a reasonable number appeared to have commonalities beyond just their shared account ID. This threshold varied depending on the account type.
    3. We deduplicated index-contact pairs identified via multiple shared accounts, retaining pertinent information from both sources.
    4. We used guarantor relationships to infer index-contact patient relationship types. For example, if the index patient was the child of the guarantor, and the contact patient was also the child of the guarantor, we classified the index-contact relationship as “siblings.”
    5. We combined this cleaned set of relationships with the existing table of relationships from the other sources, and deduplicated using the same methods as above.
  1. **Characteristics**

These relationships were treated as static and did not require a shared address. relationships from this table were coded treated as static (non-temporal). After deduplication, the combined relationship table (Family Linkage, Patient Relationship, and Shared Coverage Account) included **101,246 relationships for 44,082 unique index patients and 94,882 unique contact patients.**

1. **Address History Table**
   1. **Data Source Description**

This table contains patient address histories, including full street address, geocoded latitude and longitude, and start/end dates of the residency.

- 1. **Extraction**

We first extracted all addresses for the index patient cohort with no date restrictions. After cleaning and standardizing the addresses and geocoded coordinates (see below for details), we extracted all records for other patients sharing either exact street address or coordinates with an index patient. The initial index patient address extraction yielded **128,526 addresses for 73,531 index patients.**

- 1. **Address Cleaning/Standardization**

We standardized and cleaned addresses to improve matching accuracy by:

- - 1. Standardizing street directions, suffixes, and apartment/unit identifiers
    2. Removing all non-alphanumeric characters
    3. Nullifying invalid/placeholder text (e.g., “PLEASE UPDATE”, “9999999”)
    4. Parsing apartment/unit data into a separate field if included in the street address field
    5. Reconciling addresses with conflicting coordinates using spatial averaging
  1. **Address Date Windows**

For a shared address to reliably indicate a household or social connection, the two patients must have resided at the address at the same time. Thus, we also removed all addresses where both the start and end dates were outside the study period, and adjusted start/end dates to ensure continuous, non-overlapping address histories.

- 1. **Identification of Group Quarters and Apartments**

To avoid matching patients who shared geocoded coordinates or street address of a large institution or apartment building which would not reliably indicate a true social/household connection, we flagged addresses that appeared to be group quarters or apartment buildings. Addresses were flagged when

- - 1. They were shared by more than 12 patients, but had a small number of units (group quarters)
    2. They were shared by more than 12 patients, and had more than 2 units (apartments)
    3. Positive string searches for keywords (e.g., “shelter”, “assisted living”, “school”)
    4. Manual verification using Google Maps (manual verification also allowed us to classify the type of group quarters).
  1. **Matching Process**
     1. **Single Family Homes**

After removing addresses flagged as group quarters or apartments, we identified pairs based on exact matches of street address (including city and state) or geocoded coordinates, excluding addresses with more than 30 distinct relationships, as these were unlikely to indicate true social/household relationships. We also excluded any pairs where the index and contact patient did not share the address concurrently. This initial matching yielded **632,659 relationships for 39,903 unique index patients and 282,229 unique contact patients.**

- - 1. **Apartments**

Using the same process but adding the requirement that unit/apartment number matched, we identified an additional **227,926 relationships for 24,872 unique index patients and 99,827 unique contact patients.** Note that “apartments” here also included other residences with multiple units sharing a single street address, e.g. mobile home parks, duplexes, etc.

- - 1. **Group Quarters**

We classified group quarters into categories and retained those likely relevant for potential TB transmission (shelters, assisted living/nursing homes, boarding schools), excluding others (e.g., hotels, mailing centers). The process for matching patients was as above, with the same requirement that there be some temporal overlap; however, within this set of relationships the majority of addresses were homeless shelters (78%) and had no end date, likely due to patient transience. These were flagged for cautious interpretation. This initial matching yielded **2,044,603 relationships for 2,637 unique index patients and 97,296 unique contact patients.**

- 1. **Characteristics**

Address-based relationships required exact matching on address or geocoded coordinates with concurrent residency for at least one day. The type of group quarters was recorded where applicable.

1. **Final Relationship Table**
   1. **Deduplication**

We combined the address-based relationships with those from prior sources, deduplicating as above while preserving relevant relationship and temporal information from all sources when a relationship was identified in multiple ways. Duplicate index-contact pairs were retained when they shared multiple distinct addresses to preserve shared address dates for each distinct address. The final table contained **1,570,415 relationships for 63,717 unique index patients and 326,258 unique contact patients.** After restricting the analytic sample to include index patients tested between 2014-2022 (to enable ascertainment of contacts’ testing status with a 2-year window around this period in the dataset—i.e., 2012-2024), and assigning the first individual in a household with a positive test as the index case, the analytic sample contained **965,559 relationships for 42,009 index patients and 241,104 unique contact patients.**

- 1. **Characteristics**

Relationships identified via Family Linkage table; Patient Relationships table and Insurance Coverage table were considered static and retained explicit relationship types from each underlying source. Address-based relationships were classified as undetermined relationship type but included temporal overlap periods and group quarters type where applicable.

We assigned a “partner” relationship when the relation was self-reported as wife, husband, spouse, significant other, or “partner”. Relationships were inferred from the mother-child linkage table. When the relationship was unknown but individuals shared an address, we labeled dyads as shared household members with unspecified relationships.

1. **General Notes**

Throughout all stages, we applied consistent, rigorous cleaning and filtering decisions to maximize capture of index patient’s social, family, and household network while avoiding over-capture of connections that were not relevant to TB transmission. We maintained detailed audit records documenting exclusions and filtering decisions allowing transparent review and future replication or refinement as needed.

**
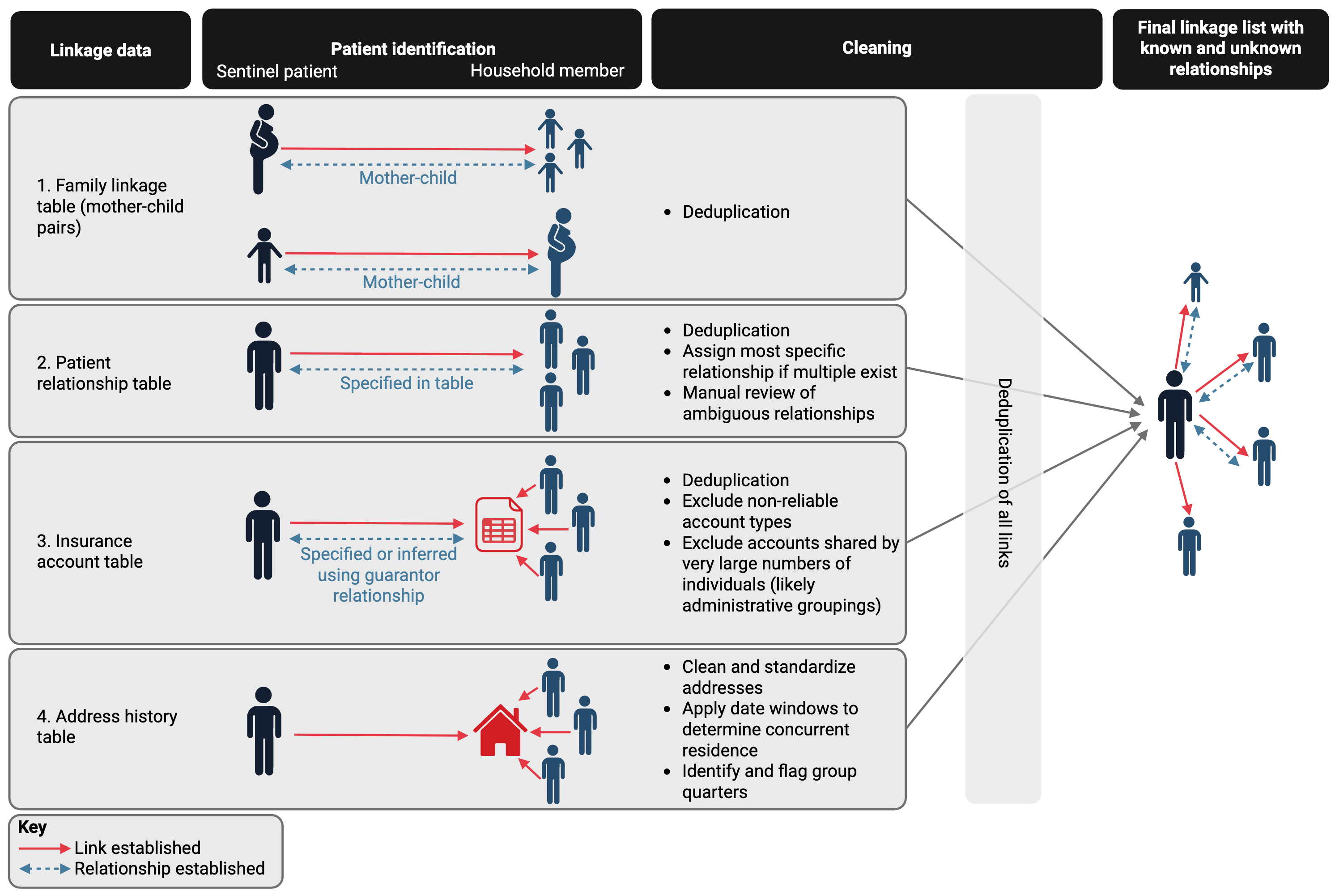
**

**Supplemental figure 1.** Schematic of the household linking strategy, illustrating data sources and data processing for each linkage type.

**Supplemental Table 1.** Categorization of reported relationships.

|  | **Categorized relationship** | | | | | | | | | |
| --- | --- | --- | --- | --- | --- | --- | --- | --- | --- | --- |
| **Reported relationship** | Child (N=29328) | Sibling  (N=2199) | Parent  (sentinel <5yo)  (N=465) | Parent  (sentinel 5-14yo)  (N=1173) | Parent  (sentinel 15+yo)  (N=8259) | Partner  (N=669) | Other relative  (N=564) | Shared household  (rel. not specified)  (N=86,767) | Missing  (N=8) | Total  (N=129432) |
| Aunt | 0 | 0 | 0 | 0 | 0 | 0 | 3 | 0 | 0 | 3 |
| Brother | 0 | 5 | 0 | 0 | 0 | 0 | 0 | 0 | 0 | 5 |
| Child | 29,213 | 0 | 0 | 0 | 0 | 0 | 0 | 259 | 0 | 29,472 |
| Cousin | 0 | 0 | 0 | 0 | 0 | 0 | 3 | 0 | 0 | 3 |
| Daughter | 26 | 0 | 0 | 0 | 0 | 0 | 0 | 0 | 0 | 26 |
| Ex-Spouse | 0 | 0 | 0 | 0 | 0 | 1 | 0 | 0 | 0 | 1 |
| Father | 0 | 0 | 30 | 20 | 16 | 0 | 0 | 0 | 0 | 66 |
| Foster child/ward | 76 | 0 | 0 | 0 | 0 | 0 | 0 | 0 | 0 | 76 |
| Foster sibling | 0 | 4 | 0 | 0 | 0 | 0 | 0 | 0 | 0 | 4 |
| Friend | 0 | 0 | 0 | 0 | 0 | 0 | 0 | 0 | 7 | 7 |
| Grandchild | 0 | 0 | 0 | 0 | 0 | 0 | 2 | 0 | 0 | 2 |
| Grandparent | 0 | 0 | 0 | 0 | 0 | 0 | 1 | 0 | 0 | 1 |
| Immediate family | 0 | 0 | 0 | 0 | 0 | 0 | 140 | 0 | 0 | 140 |
| Immediate or extended family | 0 | 0 | 0 | 0 | 0 | 0 | 9 | 0 | 0 | 9 |
| Mother | 0 | 0 | 432 | 1,152 | 8,210 | 0 | 350* | 0 | 0 | 10,144 |
| Niece/Nephew | 0 | 0 | 0 | 0 | 0 | 0 | 54 | 0 | 0 | 54 |
| Other Guardian | 0 | 0 | 0 | 0 | 0 | 0 | 2 | 0 | 0 | 2 |
| Parent | 0 | 0 | 3 | 0 | 32 | 0 | 0 | 0 | 0 | 35 |
| Partner | 0 | 0 | 0 | 0 | 0 | 3 | 0 | 0 | 0 | 3 |
| Shared household | 0 | 0 | 0 | 0 | 0 | 0 | 0 | 85,414 | 0 | 85,414 |
| Sibling | 0 | 2 | 0 | 0 | 0 | 0 | 0 | 0 | 0 | 2 |
| Sibling/step sibling | 0 | 2,184 | 0 | 0 | 0 | 0 | 0 | 0 | 0 | 2,184 |
| Significant other | 0 | 0 | 0 | 0 | 0 | 12 | 0 | 0 | 0 | 12 |
| Sister | 0 | 3 | 0 | 0 | 0 | 0 | 0 | 0 | 0 | 3 |
| Son | 13 | 0 | 0 | 0 | 0 | 0 | 0 | 0 | 0 | 13 |
| Son-in-Law | 0 | 0 | 0 | 0 | 0 | 0 | 0 | 0 | 1 | 1 |
| Spouse/Partner | 0 | 0 | 0 | 0 | 0 | 653 | 0 | 0 | 0 | 653 |
| Step parent | 0 | 0 | 0 | 1 | 1 | 0 | 0 | 0 | 0 | 2 |
| Stepsister | 0 | 1 | 0 | 0 | 0 | 0 | 0 | 0 | 0 | 1 |
| Undetermined | 0 | 0 | 0 | 0 | 0 | 0 | 0 | 1,094 | 0 | 1,093 |

*Classified as "other relative" when index age was not known

**Supplemental Table 2.** Definitions of comorbidities and TB disease.

| **Condition** | **Timing** | **Coding** |
| --- | --- | --- |
| HIV | Any time before to 2y after sentinel test date | **ICD9:** 42, 42.1, 42.9, 43.0, 43.1, 43.2, 43.3, 43.9, 44.0, 44.9  **ICD10:** B20.1, B20.2, B20.3, B20.4, B20.5, B20.6, B20.7, B20.8, B20.9, B21.0, B21.1, B21.2, B21.3, B21.7, B21.8, B21.9, B22.0, B22.1, B22.2, B22.7, B22.0, B22.1, B22.2, B22.7, B23.0, B23.1, B23.2, B23.8, B24.0, Z21 |
| Organ transplant | Any time before to 2y after sentinel test date | **ICD9:** V42.*, V58.44* **ICD10:** Z94, Z48.298 |
| Other immunocompromising conditions | 2y before to 2y after sentinel test date (i.e., limited to immunocompromising conditions contemporaneous with sentinel TB infection testing) | **ICD10:** L73.2 **Prescription:** Abatacept, abrocitinib, adalimumab, alemtuzumab, anakinra, baricitinib, brodalumab, canakinumab, certolizumab, deucravacitinib, emapalumab, etanercept, golimumab, guselkumab, inebilizumab, infliximab, ixekizumab, rilonacept, risankizumab, rituximab, ruxolitinib, sarilumab, satralizumab, secukinumab, spesolimab, tildrakizumab, tocilizumab, tofacitinib, upadacitinib, ustekinumab, vedolizumab, methylprednisolone, prednisone >13 days, prednisolone >13 days |
| TB exposure | Any time | **ICD9:** V01.1 **ICD10:** Z20.1 |
| End stage renal disease | Any time before to 2y after sentinel test date | **ICD9:** 996.56, 996.68, 996.73, 458.21, V45.1, V56, V56.8, 39.95, 54.98 **ICD10:** N18.6 |
| Chronic kidney disease | Any time before to 2y after sentinel test date | **ICD9:** 582, 583, 585, 586, 587 **ICD10:** N18* |
| Diabetes | Any time before to 2y after sentinel test date | **ICD9:** 250.* on 2 encounters **ICD10:** E10*, E11* |
| Inflammatory bowel disease | Any time before to 2y after sentinel test date | **ICD9:** 555.*, 556.* **ICD10:** K50.0*, K50.1*, K50.8*, K50.9*, K51.0*, K51.2*, K51.3*, K51.4*, K51.5*, K51.8*, K51.9 |
| Other rheumatologic conditions | Any time before to 2y after sentinel test date | **ICD9:** 710.0, 710.1, 710.2, 710.3, 710.4, 710.8, 710.9, 446, 725 **ICD10:** M08.1, M45.*, M04.2, M08.*, M05.*, M06.*, M32.1*, M32.8, M32.9 |
| Hepatic failure | Any time before to 2y after sentinel test date | **ICD10:** K72* |
| History of latent tuberculous infection | Any time before sentinel test date | **ICD9:** 795.51, 795.52 **ICD10:** R76.11, R76.12, Z22.7 |
| TB disease [1,2] | Any time | **Definition 1:** Pyrazinamide + 2 of (isoniazid, rifampicin, ethambutol, capreomycin, kanamycin, streptomycin, cycloserine, paraaminosalicylic acid, clofazimine, levofloxacin, moxifloxacin, linezolid) OR Rifampin + isoniazid + ethambutol + (levofloxacin or moxifloxacin)  OR  **Definition 2:** 1 of (ICD9 010.0-018.9) + 3 of (pyrazinamide, isoniazid, rifampicin, ethambutol, capreomycin, kanamycin, streptomycin, cycloserine, paraaminosalicylic acid, clofazimine, levofloxacin, moxifloxacin) within 60 days  OR  1 of (ICD10 A15x, A16x, A17x, A18x, A19x) + 3 of (pyrazinamide, isoniazid, rifampicin, ethambutol, capreomycin, kanamycin,  streptomycin, cycloserine, paraaminosalicylic acid, clofazimine, levofloxacin, moxifloxacin, linezolid) within 60 days |
| TB infection treatment [3] | Within 1y of positive test | **Prescription:** (isoniazid alone) OR (isoniazid + rifampin) OR (isoniazid + rifapentine) OR (isoniazid + rifabutin) OR (rifampin alone) OR rifabutin alone WITHOUT 1 of (pyrazinamide, ethambutol, cycloserine, ethionamide, clofazimine, linezolid)   **Prescription:** If rifampin alone, then:  WITHOUT  1 of (duration <30 days, duration >365 days)  **Prescription:** If rifampin monotherapy duration >30 days and <365 days, then  WITHOUT  1 of (**ICD10:** A79.82, A44.*, A79.0, A23.*, L29.8, L29.9, I33.0, T82.6*, T82.7*, L73.2, A30.*, Z20.811, A31.*, B95.61, B95.62, B95.7, B95.8; M86.*; M00.0*, T84.5*, T84.6*, T84.7*; G00.3, G04.2, G06.*, G07, G08; R78.81; T85.7*; T81.4*; S91.*; L97.*; L89.*, Z22.338 **ICD9:** 082.49, 088.0, 023.x, 421.0, 421.1, 421.9, 424.90, 424.91, 424.99, 705.83, 030.x, V01.84, 038.11, 038.12, 041.11, 041.12, 041.19, 482.41, 482.42. V02.53, V02.54, 041.01) |

**Abbreviations:** ICD – International Classification of Diseases; HIV – Human Immunodeficiency Virus; TB – tuberculosis; TST – tuberculin skin test**;** y – years

References:

1. El Halabi J, Palmer N, McDuffie M, et al. Measuring health-care delays among privately insured patients with tuberculosis in the USA: an observational cohort study. The Lancet Infectious diseases **2021**; 21(8): 1175-83.

2. Calderwood MS, Platt R, Hou X, et al. Real-time surveillance for tuberculosis using electronic health record data from an ambulatory practice in eastern Massachusetts. Public Health Rep **2010**; 125(6): 843-50.

3. Vonnahme LA, Raykin J, Jones M, et al. Using Electronic Health Record Data to Measure the Latent Tuberculosis Infection Care Cascade in Safety-Net Primary Care Clinics. AJPM Focus **2023**; 2(4): 100148.

**Supplemental Table 3.** Initial patient and household member characteristics associated with household member testing from any timbe before to two years after the first initial patient positive test.

|  | Not tested (N=95618) | Tested (N=33814) | Univariable | | Multivariable | |
| --- | --- | --- | --- | --- | --- | --- |
|  |  |  | OR | 95%CI | aOR | 95%CI |
| Sentinel patient sex |  |  |  |  |  |  |
| Female | 63172 (66.1%) | 19091 (56.5%) | 0.67 | 0.64, 0.69 | 0.79 | 0.75, 0.82 |
| Male | 32440 (33.9%) | 14723 (43.5%) | REF |  | REF |  |
| Missing | 6 (<1%) | 0 (0.0%) |  |  |  |  |
| Household member sex |  |  |  |  |  |  |
| Female | 52737 (55.2%) | 18926 (56.0%) | 1.03 | 1.01, 1.06 | 1.08 | 1.05, 1.11 |
| Male | 42856 (44.8%) | 14886 (44.0%) | REF |  | REF |  |
| Missing | 25 (<1%) | 2 (<1%) |  |  |  |  |
| Sentinel patient age |  |  |  |  |  |  |
| <5 years old | 1459 (1.5%) | 1022 (3.0%) | 1.86 | 1.63, 2.11 | 1.39 | 1.22, 1.58 |
| 5-14 years old | 4580 (4.8%) | 2466 (7.3%) | 1.43 | 1.32, 1.54 | 1.36 | 1.26, 1.47 |
| 15-24 years old | 11905 (12.5%) | 4009 (11.9%) | 0.89 | 0.84, 0.95 | 0.94 | 0.89, 1.00 |
| 25-44 years old | 38312 (40.1%) | 14447 (42.7%) | REF |  | REF |  |
| 45-64 years old | 28166 (29.5%) | 8713 (25.8%) | 0.82 | 0.78, 0.86 | 0.88 | 0.84, 0.92 |
| 65+ years old | 8530 (8.9%) | 2556 (7.6%) | 0.79 | 0.74, 0.85 | 0.9 | 0.83, 0.97 |
| Missing | 2666 (2.8%) | 601 (1.8%) |  |  |  |  |
| Household member age |  |  |  |  |  |  |
| <5 years old | 7998 (8.4%) | 3700 (10.9%) | 1.38 | 1.32, 1.45 | 1.26 | 1.19, 1.32 |
| 5-14 years old | 17858 (18.7%) | 8750 (25.9%) | 1.46 | 1.41, 1.52 | 1.49 | 1.43, 1.55 |
| 15-24 years old | 16746 (17.5%) | 6508 (19.2%) | 1.16 | 1.12, 1.21 | 1.32 | 1.27, 1.38 |
| 25-44 years old | 27035 (28.3%) | 9049 (26.8%) | REF |  | REF |  |
| 45-64 years old | 18903 (19.8%) | 4361 (12.9%) | 0.69 | 0.66, 0.72 | 0.63 | 0.60, 0.66 |
| 65+ years old | 7078 (7.4%) | 1446 (4.3%) | 0.61 | 0.57, 0.65 | 0.47 | 0.43, 0.50 |
| Sentinel patient language |  |  |  |  |  |  |
| English | 28807 (30.1%) | 6654 (19.7%) | REF |  | REF |  |
| Spanish | 40919 (42.8%) | 10147 (30.0%) | 1.07 | 1.03, 1.12 | 1.08 | 1.02, 1.14 |
| Haitian Creole | 1376 (1.4%) | 336 (1.0%) | 1.06 | 0.92, 1.21 | 0.89 | 0.76, 1.04 |
| Other | 24473 (25.6%) | 16646 (49.2%) | 2.94 | 2.81, 3.09 | 1.51 | 1.43, 1.59 |
| Missing | 43 (<1%) | 31 (0.1%) |  |  |  |  |
| Household member language |  |  |  |  |  |  |
| English | 41407 (43.3%) | 8756 (25.9%) | REF |  | REF |  |
| Spanish | 35423 (37.0%) | 9067 (26.8%) | 1.21 | 1.17, 1.26 | 1.29 | 1.23, 1.35 |
| Haitian Creole | 1004 (1.1%) | 247 (0.7%) | 1.16 | 1.00, 1.36 | 1.26 | 1.06, 1.50 |
| Other | 16989 (17.8%) | 15643 (46.3%) | 4.35 | 4.17, 4.54 | 3.75 | 3.57, 3.94 |
| Missing | 795 (0.8%) | 101 (0.3%) |  |  |  |  |
| Sentinel patient and household Social Vulnerability Index |  |  |  |  |  |  |
| Quartile 1 | 4992 (5.2%) | 1841 (5.4%) | REF |  | REF |  |
| Quartile 2 | 11038 (11.5%) | 3838 (11.4%) | 0.94 | 0.86, 1.03 | 0.98 | 0.90, 1.07 |
| Quartile 3 | 21559 (22.5%) | 7761 (23.0%) | 0.98 | 0.90, 1.06 | 1.00 | 0.92, 1.09 |
| Quartile 4 | 52703 (55.1%) | 18754 (55.5%) | 0.96 | 0.89, 1.04 | 0.98 | 0.90, 1.06 |
| Missing | 5326 (5.6%) | 1620 (4.8%) |  |  |  |  |
| Sentinel patient HIV | 1291 (1.4%) | 324 (1.0%) | 0.71 | 0.60, 0.83 | 0.86 | 0.73, 1.01 |
| Household member HIV | 337 (0.4%) | 304 (0.9%) | 2.56 | 2.20, 3.00 | 4.67 | 3.84, 5.68 |
| Sentinel patient immunocompromising condition | 3760 (3.9%) | 970 (2.9%) | 0.72 | 0.65, 0.80 | 0.93 | 0.84, 1.04 |
| Household member immunocompromising condition | 2155 (2.3%) | 854 (2.5%) | 1.12 | 1.03, 1.22 | 1.46 | 1.33, 1.61 |
| Sentinel patient other comorbidity | 18407 (19.3%) | 4894 (14.5%) | 0.71 | 0.68, 0.74 | 0.89 | 0.85, 0.94 |
| Household member other comorbidity | 10107 (10.6%) | 2564 (7.6%) | 0.69 | 0.66, 0.73 | 1.12 | 1.06, 1.19 |
| Sentinel patient started treatment | 18688 (19.5%) | 7154 (21.2%) | 1.1 | 1.06, 1.15 | 1.16 | 1.10, 1.21 |

**Abbreviations:** IGRA – interferon gamma release assay; HIV – Human Immunodeficiency Virus; TB – tuberculosis; TST – tuberculin skin test

**Supplemental Table 4.** Initial patient and household member characteristics associated with household member testing positive if tested within two years of the first initial patient positive test.

|  | Tested negative  (N=27836) | Tested positive (N=3127) | Univariable | | Multivariable | |
| --- | --- | --- | --- | --- | --- | --- |
|  |  |  | OR | 95%CI | aOR | 95%CI |
| Sentinel patient sex |  |  |  |  |  |  |
| Female | 15472 (55.6%) | 1737 (55.5%) | 1.00 | 0.92, 1.09 | 1.13 | 1.03, 1.23 |
| Male | 12364 (44.4%) | 1390 (44.5%) | REF |  | REF |  |
| Household member sex |  |  |  |  |  |  |
| Female | 15763 (56.6%) | 1646 (52.6%) | 0.85 | 0.79, 0.91 | 0.76 | 0.71, 0.83 |
| Male | 12071 (43.4%) | 1481 (47.4%) | REF |  | REF |  |
| Missing | 2 (<1%) | 0 (0.0%) |  |  |  |  |
| Sentinel patient age |  |  |  |  |  |  |
| <5 years old | 795 (2.9%) | 117 (3.7%) | 1.68 | 1.34, 2.10 | 1.62 | 1.26, 2.07 |
| 5-14 years old | 1929 (6.9%) | 367 (11.7%) | 2.17 | 1.87, 2.52 | 2.69 | 2.28, 3.17 |
| 15-24 years old | 3212 (11.5%) | 445 (14.2%) | 1.58 | 1.39, 1.80 | 1.48 | 1.29, 1.71 |
| 25-44 years old | 12086 (43.4%) | 1059 (33.9%) | REF |  | REF |  |
| 45-64 years old | 7242 (26.0%) | 786 (25.1%) | 1.24 | 1.11, 1.38 | 0.89 | 0.80, 1.01 |
| 65+ years old | 2097 (7.5%) | 302 (9.7%) | 1.64 | 1.42, 1.90 | 0.87 | 0.73, 1.03 |
| Missing | 475 (1.7%) | 51 (1.6%) |  |  |  |  |
| Household member age |  |  |  |  |  |  |
| <5 years old | 3273 (11.8%) | 112 (3.6%) | 0.22 | 0.18, 0.27 | 0.18 | 0.14, 0.22 |
| 5-14 years old | 7329 (26.3%) | 339 (10.8%) | 0.3 | 0.26, 0.34 | 0.26 | 0.22, 0.30 |
| 15-24 years old | 5480 (19.7%) | 496 (15.9%) | 0.59 | 0.52, 0.66 | 0.6 | 0.53, 0.67 |
| 25-44 years old | 7364 (26.5%) | 1139 (36.4%) | REF |  | REF |  |
| 45-64 years old | 3308 (11.9%) | 773 (24.7%) | 1.51 | 1.36, 1.67 | 1.59 | 1.42, 1.79 |
| 65+ years old | 1082 (3.9%) | 268 (8.6%) | 1.6 | 1.38, 1.86 | 1.65 | 1.39, 1.97 |
| Sentinel patient language |  |  |  |  |  |  |
| English | 5298 (19.0%) | 544 (17.4%) | REF |  | REF |  |
| Spanish | 8233 (29.6%) | 694 (22.2%) | 0.82 | 0.72, 0.93 | 0.91 | 0.78, 1.06 |
| Haitian Creole | 267 (1.0%) | 48 (1.5%) | 1.75 | 1.28, 2.39 | 1.59 | 1.11, 2.28 |
| Other | 14011 (50.3%) | 1838 (58.8%) | 1.28 | 1.15, 1.42 | 1.18 | 1.01, 1.36 |
| Missing | 27 (0.1%) | 3 (0.1%) |  |  |  |  |
| Household member language |  |  |  |  |  |  |
| English | 7043 (25.3%) | 566 (18.1%) | REF |  | REF |  |
| Spanish | 7362 (26.4%) | 676 (21.6%) | 1.14 | 1.01, 1.29 | 1.19 | 1.03, 1.37 |
| Haitian Creole | 199 (0.7%) | 42 (1.3%) | 2.63 | 1.85, 3.72 | 1.92 | 1.28, 2.87 |
| Other | 13150 (47.2%) | 1830 (58.5%) | 1.73 | 1.56, 1.92 | 1.64 | 1.42, 1.90 |
| Missing | 82 (0.3%) | 13 (0.4%) |  |  |  |  |
| Sentinel patient and household Social Vulnerability Index |  |  |  |  |  |  |
| Quartile 1 | 1537 (5.5%) | 187 (6.0%) | REF |  | REF |  |
| Quartile 2 | 3124 (11.2%) | 373 (11.9%) | 0.98 | 0.80, 1.20 | 1.05 | 0.86, 1.30 |
| Quartile 3 | 6409 (23.0%) | 660 (21.1%) | 0.85 | 0.70, 1.02 | 0.94 | 0.77, 1.15 |
| Quartile 4 | 15441 (55.5%) | 1780 (56.9%) | 0.95 | 0.80, 1.13 | 1.09 | 0.91, 1.31 |
| Missing | 1325 (4.8%) | 127 (4.1%) |  |  |  |  |
| Sentinel patient HIV | 271 (1.0%) | 30 (1.0%) | 0.99 | 0.66, 1.46 | 1.15 | 0.73, 1.82 |
| Household member HIV | 278 (1.0%) | 19 (0.6%) | 0.61 | 0.38, 0.97 | 0.41 | 0.25, 0.68 |
| Sentinel patient immunocompromising condition | 804 (2.9%) | 75 (2.4%) | 0.83 | 0.65, 1.06 | 0.94 | 0.72, 1.23 |
| Household member immunocompromising condition | 692 (2.5%) | 77 (2.5%) | 0.99 | 0.78, 1.26 | 0.84 | 0.65, 1.09 |
| Sentinel patient other comorbidity | 3952 (14.2%) | 499 (16.0%) | 1.15 | 1.03, 1.28 | 1.2 | 1.05, 1.37 |
| Household member other comorbidity | 1954 (7.0%) | 446 (14.3%) | 2.2 | 1.97, 2.46 | 1.24 | 1.09, 1.42 |
| Sentinel patient started treatment | 6007 (21.6%) | 644 (20.6%) | 0.94 | 0.85, 1.04 | 0.94 | 0.84, 1.05 |

**Abbreviations:** IGRA – interferon gamma release assay; HIV – Human Immunodeficiency Virus; TB – tuberculosis; TST – tuberculin skin test

**Supplemental Table 5.** Initial patient and household member characteristics associated with household members being prescribed treatment within 1 year of testing positive.

|  | Not prescribed treatment (N=2474) | Prescribed treatment (N=641) | Univariable | | Multivariable | |
| --- | --- | --- | --- | --- | --- | --- |
|  |  |  | OR | 95%CI | aOR | 95%CI |
| Sentinel patient sex |  |  |  |  |  |  |
| Female | 1370 (55.4%) | 360 (56.2%) | 1.03 | 0.85, 1.25 | 1.03 | 0.82, 1.29 |
| Male | 1104 (44.6%) | 281 (43.8%) | REF |  | REF |  |
| Household member sex |  |  |  |  |  |  |
| Female | 1318 (53.3%) | 321 (50.1%) | 0.88 | 0.74, 1.05 | 0.73 | 0.59, 0.90 |
| Male | 1156 (46.7%) | 320 (49.9%) | REF |  | REF |  |
| Missing |  |  |  |  |  |  |
| Sentinel patient age |  |  |  |  |  |  |
| <5 years old | 91 (3.7%) | 25 (3.9%) | 1.18 | 0.69, 2.01 | 1.05 | 0.62, 1.81 |
| 5-14 years old | 279 (11.3%) | 86 (13.4%) | 1.32 | 0.94, 1.85 | 1.07 | 0.72, 1.59 |
| 15-24 years old | 338 (13.7%) | 105 (16.4%) | 1.33 | 1.00, 1.78 | 1.18 | 0.85, 1.64 |
| 25-44 years old | 856 (34.6%) | 200 (31.2%) | REF |  | REF |  |
| 45-64 years old | 626 (25.3%) | 159 (24.8%) | 1.09 | 0.85, 1.40 | 0.99 | 0.73, 1.34 |
| 65+ years old | 246 (9.9%) | 55 (8.6%) | 0.96 | 0.66, 1.38 | 1.1 | 0.68, 1.78 |
| Missing | 38 (1.5%) | 11 (1.7%) |  |  |  |  |
| Household member age |  |  |  |  |  |  |
| <5 years old | 81 (3.3%) | 31 (4.8%) | 1.66 | 1.04, 2.66 | 1.32 | 0.72, 2.44 |
| 5-14 years old | 256 (10.3%) | 79 (12.3%) | 1.34 | 0.97, 1.85 | 1.31 | 0.91, 1.90 |
| 15-24 years old | 406 (16.4%) | 89 (13.9%) | 0.95 | 0.72, 1.25 | 0.88 | 0.64, 1.20 |
| 25-44 years old | 922 (37.3%) | 212 (33.1%) | REF |  | REF |  |
| 45-64 years old | 588 (23.8%) | 184 (28.7%) | 1.36 | 1.08, 1.71 | 1.33 | 0.99, 1.79 |
| 65+ years old | 221 (8.9%) | 46 (7.2%) | 0.91 | 0.64, 1.29 | 0.88 | 0.55, 1.39 |
| Sentinel patient language |  |  |  |  |  |  |
| English | 400 (16.2%) | 143 (22.3%) | REF |  | REF |  |
| Spanish | 508 (20.5%) | 185 (28.9%) | 1.02 | 0.78, 1.33 | 0.66 | 0.41, 1.06 |
| Haitian Creole | 38 (1.5%) | 10 (1.6%) | 0.74 | 0.35, 1.53 | 0.83 | 0.31, 2.20 |
| Other | 1527 (61.7%) | 301 (47.0%) | 0.55 | 0.43, 0.71 | 0.66 | 0.47, 0.94 |
| Missing | 1 (<1%) | 2 (0.3%) |  |  |  |  |
| Household member language |  |  |  |  |  |  |
| English | 423 (17.1%) | 141 (22.0%) | REF |  | REF |  |
| Spanish | 484 (19.6%) | 190 (29.6%) | 1.18 | 0.90, 1.54 | 1.44 | 0.88, 2.34 |
| Haitian Creole | 35 (1.4%) | 7 (1.1%) | 0.6 | 0.26, 1.40 | 1.07 | 0.43, 2.70 |
| Other | 1520 (61.4%) | 302 (47.1%) | 0.6 | 0.47, 0.76 | 0.86 | 0.61, 1.20 |
| Missing | 12 (0.5%) | 1 (0.2%) |  |  |  |  |
| Sentinel patient and household Social Vulnerability Index |  |  |  |  |  |  |
| Quartile 1 | 160 (6.5%) | 26 (4.1%) | REF |  | REF |  |
| Quartile 2 | 298 (12.0%) | 73 (11.4%) | 1.51 | 0.92, 2.47 | 1.54 | 0.91, 2.61 |
| Quartile 3 | 518 (20.9%) | 139 (21.7%) | 1.65 | 1.04, 2.63 | 1.99 | 1.21, 3.27 |
| Quartile 4 | 1403 (56.7%) | 371 (57.9%) | 1.63 | 1.04, 2.54 | 1.78 | 1.11, 2.85 |
| Missing | 95 (3.8%) | 32 (5.0%) |  |  |  |  |
| Sentinel patient HIV | 24 (1.0%) | 6 (0.9%) | 0.96 | 0.40, 2.32 | 0.75 | 0.19, 2.93 |
| Household member HIV | 15 (0.6%) | 4 (0.6%) | 1.03 | 0.34, 3.11 | 0.95 | 0.20, 4.48 |
| Sentinel patient immunocompromising condition | 56 (2.3%) | 17 (2.7%) | 1.18 | 0.65, 2.13 | 0.97 | 0.47, 2.03 |
| Household member immunocompromising condition | 67 (2.7%) | 10 (1.6%) | 0.57 | 0.29, 1.11 | 0.64 | 0.26, 1.55 |
| Sentinel patient other comorbidity | 375 (15.2%) | 123 (19.2%) | 1.33 | 1.04, 1.70 | 1.47 | 1.07, 2.03 |
| Household member other comorbidity | 341 (13.8%) | 104 (16.2%) | 1.21 | 0.95, 1.54 | 0.97 | 0.70, 1.35 |
| Sentinel patient started treatment | 281 (11.4%) | 357 (55.7%) | 9.81 | 7.94, 12.12 | 9.68 | 7.71, 12.16 |

**Abbreviations:** IGRA – interferon gamma release assay; HIV – Human Immunodeficiency Virus; TB – tuberculosis; TST – tuberculin skin test

**Supplemental Figure 2.** Time to household member TB infection testing in relation to initial patient first positive test date. The blue curve represents the cumulative number of household members tested. The red dashed line indicates the two-year cutoff used in the primary outcome for this study.

**Supplemental Figure 3.** Time between sentinel patient positive test and household member positive test, for those household members with a positive test. The blue curve represents the cumulative number of household members testing positive. The red dashed line indicates the two-year cutoff used in the primary outcome for this study.

**Supplemental Figure 4.**  Time between household member positive test date and treatment prescription date, for those prescribed treatment within 1 year of the positive test (the primary treatment outcome). The blue curve represents the cumulative number of household members prescribed treatment. The red dashed line indicates the one-year cutoff used in the primary outcome for this study.

**Supplemental figure 5.** Marginal predicted probabilities of a household member being tested, having a positive test, and starting treatment. Probabilities are derived from outputs of the primary outcome multivariable logistic regression models.

**Supplemental Figure 6.** Characteristics of sentinel patients, household members, and households associated with household member testing within two years of the sentinel patient, test positivity within two years of the sentinel patient, and treatment prescription within 1 year of positive test, excluding sentinel patient-household member links when both tested positive on the same day.

**Abbreviations:** ICC – immunocompromising condition; IGRA – interferon gamma release assay; incid. crty – “incident country”; HHM – household member; Ref – reference; TB – tuberculosis; TST – tuberculin skin test

**Supplemental Figure 7.** Characteristics of sentinel patients, household members, and households associated with household member testing any time before or after the sentinel patient, test positivity any time after the sentinel patient, and treatment prescription within 1 year of positive test.

**Abbreviations:** ICC – immunocompromising condition; IGRA – interferon gamma release assay; incid. crty – “incident country”; HHM – household member; Ref – reference; TB – tuberculosis; TST – tuberculin skin test

**Supplemental Figure 8.** Characteristics of sentinel patients, household members, and households associated with household member testing within two years of the sentinel patient, test positivity within two years of the sentinel patient, and treatment prescription within 1 year of positive test, restricted to linkages established through shared addresses.

**Abbreviations:** ICC – immunocompromising condition; IGRA – interferon gamma release assay; HHM – household member; Ref – reference; TB – tuberculosis; TST – tuberculin skin test

**Supplemental Figure 9.** Characteristics of sentinel patients, household members, and households associated with household member testing within two years of the sentinel patient, test positivity within two years of the sentinel patient, and treatment prescription within 1 year of positive test, restricted to linkages established through non-address administrative data.

**Abbreviations:** ICC – immunocompromising condition; IGRA – interferon gamma release assay; HHM – household member; Ref – reference; TB – tuberculosis; TST – tuberculin skin test

**Supplemental Figure 10.** Characteristics of sentinel patients, household members, and households associated with household member testing within two years of the sentinel patient, test positivity within two years of the sentinel patient, and treatment prescription within 1 year of positive test, including self-reported (complete case) and imputed birth in a country with elevated TB incidence (>10 cases/100,000 population).

**Abbreviations:** ICC – immunocompromising condition; IGRA – interferon gamma release assay; incid. crty – “incident country”; HHM – household member; Ref – reference; TB – tuberculosis; TST – tuberculin skin test

**Supplemental Figure 11.** Characteristics of sentinel patients, household members, and households associated with household member testing within two years of the sentinel patient, test positivity within two years of the sentinel patient, and treatment prescription within 1 year of positive test, using hierarchical mixed effects models. The models for HHM testing positive and treatment prescription include clustering at the level of sentinel patients, nested within the level of clinics at which the sentinel patient was tested. The model for HHM testing includes clustering at the level of the clinic where the sentinel patient was tested only, due to non-convergence of the multi-level model.

**Abbreviations:** ICC – immunocompromising condition; IGRA – interferon gamma release assay; HHM – household member; Ref – reference; TB – tuberculosis; TST – tuberculin skin test


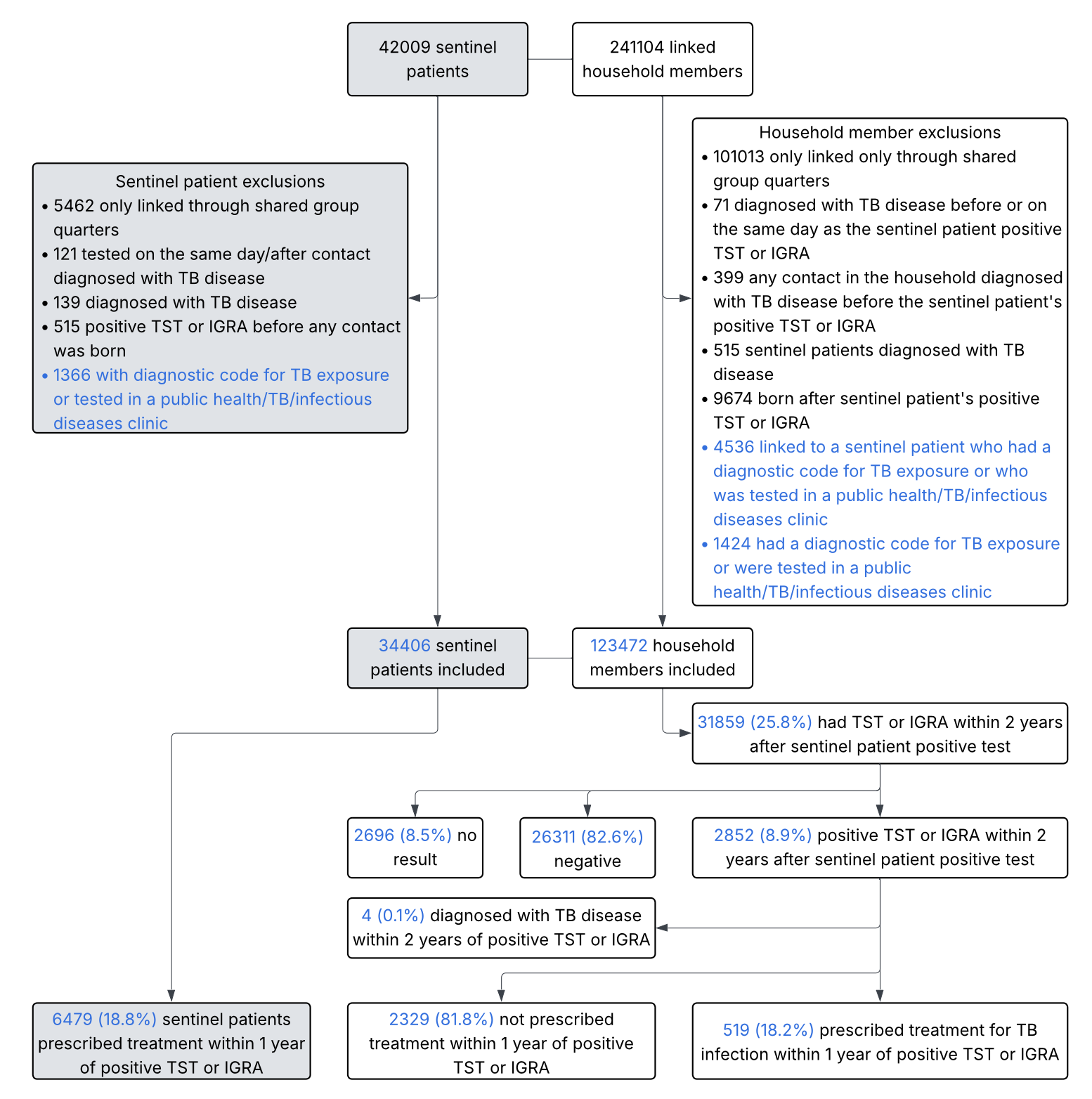
 **Supplemental figure 12.** Consort diagram and care cascade outcomes for household members, after applying rigorous exclusion of individuals potentially identified through contact tracing.

**Supplemental Figure 13.** Characteristics of sentinel patients, household members, and households associated with household member testing within two years of the sentinel patient, test positivity within two years of the sentinel patient, and treatment prescription within 1 year of positive test, after applying rigorous exclusion of individuals potentially identified through contact tracing.

**Abbreviations:** ICC – immunocompromising condition; IGRA – interferon gamma release assay; HHM – household member; Ref – reference; TB – tuberculosis; TST – tuberculin skin test


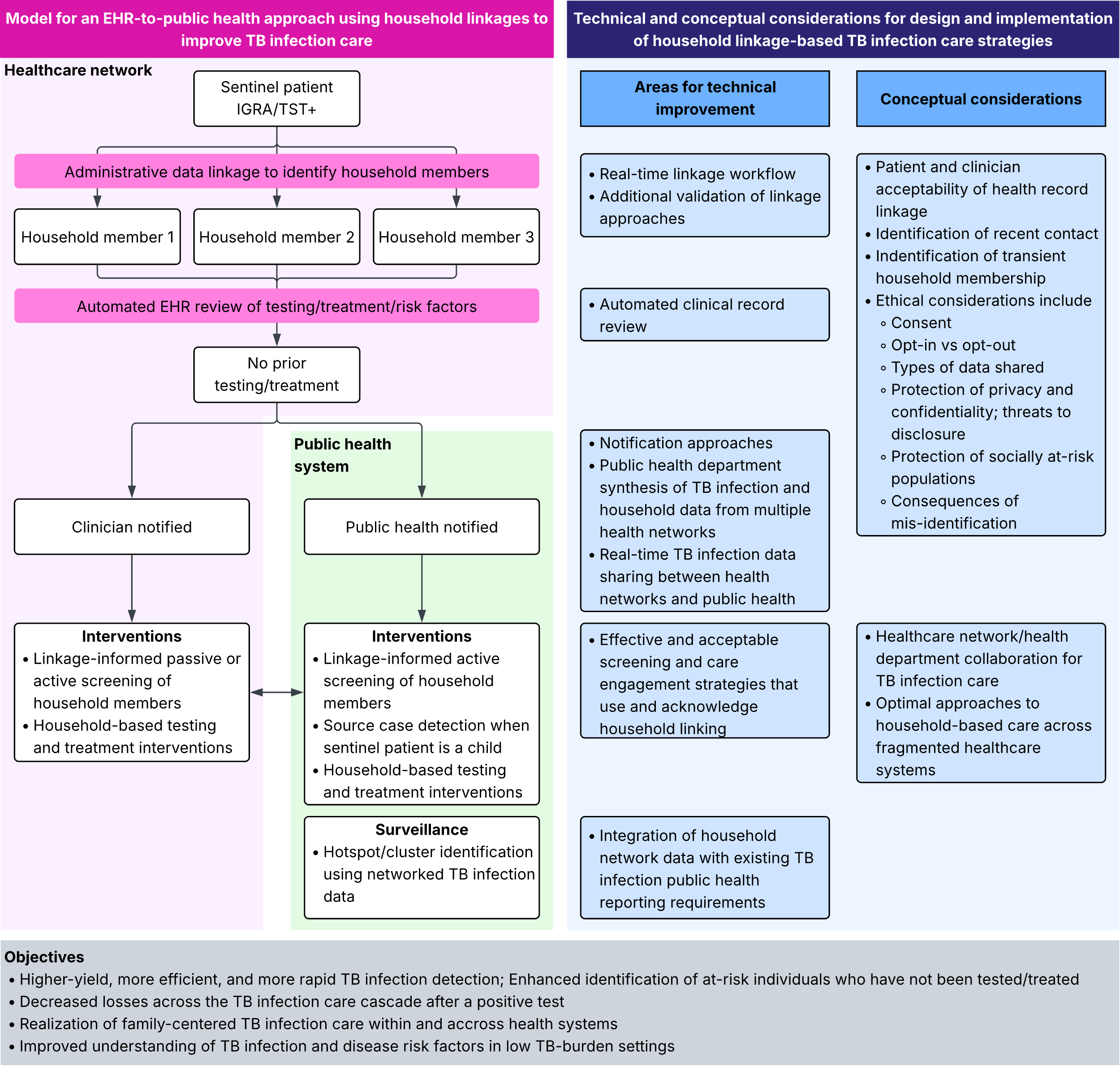


**Supplemental Figure 14.** A conceptual model of how household linking could be integrated into clinical and public health TB infection care. The pink and green panels illustrate a potential EHR-to-public health data and intervention approach. The blue panel identifies key technical and conceptual considerations development of household linking-based TB infection approaches.

**Abbreviations:** EHR – electronic health record; IGRA – interferon gamma release assay; TB – tuberculosis; TST – tuberculin skin test
